# Archaeal ancestors of eukaryotes: not so elusive any more

**DOI:** 10.1186/s12915-015-0194-5

**Published:** 2015-10-05

**Authors:** Eugene V. Koonin

**Affiliations:** National Center for Biotechnology Information, National Library of Medicine, National Institutes of Health, Bethesda, MD 20894 USA

## Abstract

The origin of eukaryotes is one of the hardest problems in evolutionary biology and sometimes raises the ominous specter of irreducible complexity. Reconstruction of the gene repertoire of the last eukaryotic common ancestor (LECA) has revealed a highly complex organism with a variety of advanced features but no detectable evolutionary intermediates to explain their origin. Recently, however, genome analysis of diverse archaea led to the discovery of apparent ancestral versions of several signature eukaryotic systems, such as the actin cytoskeleton and the ubiquitin network, that are scattered among archaea. These findings inspired the hypothesis that the archaeal ancestor of eukaryotes was an unusually complex form with an elaborate intracellular organization. The latest striking discovery made by deep metagenomic sequencing vindicates this hypothesis by showing that in phylogenetic trees eukaryotes fall within a newly identified archaeal group, the Lokiarchaeota, which combine several eukaryotic signatures previously identified in different archaea. The discovery of complex archaea that are the closest living relatives of eukaryotes is most compatible with the symbiogenetic scenario for eukaryogenesis.

A recent discovery enabled by single-cell genomics technology seems to be a huge step towards understanding the origin of eukaryotes [1, 2]. To explain why this appears to be the case, I discuss here the formidable difficulty of the problem, the previous salient observations and the proposed solutions.

A eukaryotic cell is a strikingly complex macromolecular aggregate by any account, but specifically when compared with archaeal and bacterial cells. To begin with, a typical eukaryotic cell has a three to four orders of magnitude larger volume than most bacteria and archaea [3–5]. This size difference translates into a difference in the physical principles of cell functioning: unlike most bacteria and archaea in which proteins, nucleic acids and small molecules diffuse more or less freely, the intracellular space in eukaryotes is fully compartmentalized so that molecules are distributed through specialized transport mechanisms [6, 7]. The compartmentalization and transport are supported by the elaborate system of intracellular membranes which includes the membrane of the eponymous eukaryotic organelle, the nucleus, and by an advanced cytoskeleton that consists of actin filaments and tubulin microtubules and includes numerous additional, dedicated proteins. Crucially, the great majority of eukaryotes possess the power-producing organelles, the mitochondria or their derivatives, that are now commonly accepted to have evolved from α-proteobacteria by endosymbiosis [8, 9]. Although some unicellular eukaryotes lack mitochondria, evolutionary reconstructions clearly point to secondary loss in all amitochondrial groups [10, 11].

Thus, eukaryotes show a qualitatively different level of cellular organization from that of archaea and bacteria, and there are no detectable evolutionary intermediates. Comparative analysis of eukaryotic cells and genomes indicates that the signature advanced functional systems of the eukaryotic cells were already present in the last eukaryotic common ancestor (LECA). These ancestral features include the actin and tubulin-based forms of cytoskeleton, the nuclear pore, the spliceosome, and the ubiquitin signaling network, to mention only several aspects of the inherent organizational complexity of eukaryotic cells [12–16]. The emergence of these fundamental facets of advanced cellular organization presents a challenge of such scale that Darwin’s famous scenario for the evolution of the eye looks like a straightforward solution to an easy problem. To some, the enigma of eukaryogenesis can appear so perplexing that the infamous concept of ‘irreducible complexity’ has sneaked into the scientific mainstream [17], although debunking of these ideas has not been long in coming [18]. Below I discuss the recent advances in evolutionary genomics that make the origin of eukaryotes much less mysterious than it appeared even recently.

## Phylogenetic position of the eukaryotes: sister group to archaea?

**Fig. 1:**
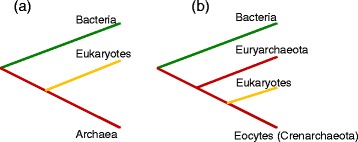
Schematic phylogenetic trees reflecting the archaeal and eocyte ancestry of eukaryotes. **a** The three-domain archaeal tree. **b** The two-domain eocyte tree

Molecular phylogenetics and phylogenomics offer a complementary perspective on the origin of eukaryotes. The standard ‘tree of life’, based initially on the sequences of 16S rRNA and subsequently on the sequences of other universal genes, such as protein components of the translation and transcription systems, unequivocally identifies the ancestry of the information-processing systems of eukaryotes as archaeal. The early versions of the tree in the standard textbooks had eukaryotes as the sister group of archaea, to the exclusion of bacteria [19–22]. However, an alternative phylogenetic method applied to the same 16S rRNAs has suggested a different, so-called eocyte tree topology [23, 24] (Fig. 1). In the eocyte tree, eukaryotes form a clade within the archaeal branch, as the sister group to the ‘eocytes’, the archaeal phylum that is currently known as Crenarchaeota [23–26]. Subsequent phylogenetic studies have reached various conclusions on the relationships between eukaryotes and archaea. Depending on the data set and the phylogenetic methodology, support has been reported for the standard placement of eukaryotes as a sister group to archaea, the eocyte topology, or various positions of the eukaryotes within the phylum Euryarchaeota, which includes mostly methanogens and halophiles [27]. Furthermore, phylogenomic analysis of multiple eukaryotic genes of archaeal provenance has pointed to their likely origins from different groups of archaea. Such findings seem to be most compatible with extensive horizontal gene transfer between the major groups of archaea, although artifacts and biases, caused in particular by differences in the characteristic evolutionary rates of these groups, could be responsible for some of the observations [28].

**Fig. 2:**
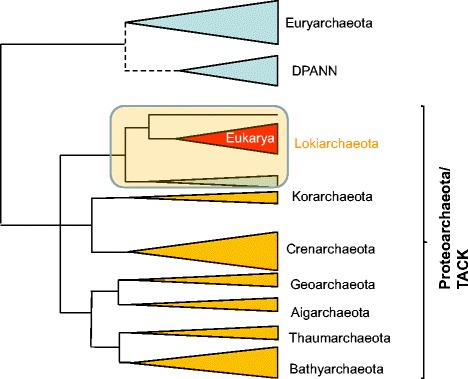
A schematic evolutionary tree of the archaea: Proteoarchaeota, Lokiarchaeota and the likely origin of eukaryotes. DPANN is the proposed archaeal superphylum that comprises Nanoarchaeota and other archaea with small genomes [33]. The tree topology is from [34] except that the DPANN branch was tentatively (as indicated by the dotted line) moved from a basal position to the stem of the Euryarchaeota on the basis of other phylogenetic analyses suggesting this [41, 42, 74]. The position of the Lokiarchaeota is from [1]. In addition to the eukaryotes, the Lokiarchaeota branch includes three distinct lineages one of which is the sister group to the eukaryotes. The size of the triangles roughly reflects the diversity of the respective groups

The uncertainty of the phylogenetic position of eukaryotes with respect to the archaea resulted from conflicting placements obtained with different methods and datasets and even a declaration of a “phylogenomic impasse” [29]. Ironically, however, shortly after the impasse was declared, progress became apparent due, above all, to the discovery of new archaeal phyla such as Korarchaeota [30], Thaumarchaeota and Aigarchaeota [31]. The latest, extensive metagenomic and single-cell genomics studies have led to a veritable ‘bonanza’ of putative new archaeal phyla [32–35] (Fig. 2). Several independent phylogenies of multiple conserved genes have consistently supported the monophyly of a deeply rooted archaeal “TACK” superphylum, named after its constituent phyla, Thaumarchaeota, Aigarchaeota, Crenarchaeota and Korarchaeota [36–40], and also provisionally designated the new kingdom Proteoarchaeota [41, 42]. A subsequent comprehensive phylogenetic study has suggested that the Proteoarchaeota additionally includes two novel phyla, Bathyarchaeota and Geoarchaeota [34] (an alternative analysis has suggested inclusion of Geoarchaeota into Crenarchaeota [43]; Fig. 2).

The discovery of the new archaeal phyla and the putative kingdom Proteoarchaeota stimulated renewed phylogenomic effort on elucidation of the archaeal ancestry of eukaryotes. Two independent, thorough phylogenetic analyses of rRNA and universal protein-coding genes demonstrated significant support for the affinity of eukaryotes with Proteoarchaeota but not with any specific lineage thereof [37, 44, 45], whereas another study placed eukaryotes within the Proteoarchaeota, as a sister group to Thaumarchaeota [46]. These results suggest an exit from the aforementioned impasse by indicating that eukaryotes most likely evolved from within the archaea, in accord with an ‘extended eocyte hypothesis’ [47]. Nevertheless, the conclusions of these phylogenomic analyses once again heavily depend on the data sets and methods employed, and arguably fall short of conclusively resolving the evolutionary relationship between archaea and eukaryotes.

## The chimeric nature of eukaryotic genomes and scenarios of eukaryogenesis

Regardless of the method employed, phylogenomic analysis of eukaryotic genes with homologs in bacteria and/or archaea reveals a fundamental split into genes of archaeal provenance and those of bacterial provenance. The ‘archaeal’ class includes primarily genes involved in information transmission whereas the ‘bacterial’ genes represent the ‘operational’ category, in particular metabolic enzymes, transporters and signal transduction systems. Notably, the ‘bacterial’ genes outnumber the ‘archaeal’ genes about twofold, indicative of a major contribution of bacteria to the genetic composition of eukaryotes [28, 48]. Given the apparent rarity of recent acquisitions of bacterial genes by eukaryotes, it appears likely that most of that contribution comes from the massive transfer of the (proto)mitochondrial genes to the nuclear genome, although relatively few genes can be traced specifically to α-proteobacteria. However, the complexity of the α-proteobacterial pangenome has made it impossible to infer the gene complement of the proto-mitochondrial endosymbiont with any precision, and could account for the apparent heterogeneity of the bacterial heritage of eukaryotes [49, 50]. A recent analysis of the relative age of the ‘bacterial’ genes in eukaryotes using a comprehensive set of genomes and advanced comparative-genomic and phylogenetic methods indeed suggests that, apart from the chloroplast-derived genes in plants and algae, the overwhelming majority of these genes have been acquired in a single sweep which is thought to be the influx from the primary endosymbiont [51].

The preponderance of genes of bacterial origin in eukaryotes begs the question: why are eukaryotes usually (even in current biology textbooks) viewed as a sister group of archaea (or possibly, eocytes) and not of α-proteobacteria? I contend that there is indeed no justification for this view, and the only consistent characterization of the evolutionary status of eukaryotes is as archaeo-bacterial chimeras. That said, not all genes are equal, and the archaeal heritage of eukaryotes includes most of the genes that are universal to the eukaryotic organisms or to all cellular life forms and are highly conserved in sequence [51, 52]. This set of (predominantly) informational genes reflects the vertical trend in the evolution of life far better than any other genes and accordingly is best suitable for the construction of the “tree of life” [53]. Nevertheless, the history of life is by no account reducible to the phylogeny of informational genes [54, 55], for which the chimeric origin and composition of the eukaryotic genome is arguably the best case in point.

Taking into account the apparent acquisition of the endosymbiont prior to LECA, the scenarios of eukaryogenesis split into two groups according to the postulated nature of the host [10]. In the first group of hypotheses, the host is envisioned as a primitive, amitochondrial, unicellular, phagotrophic eukaryote [56, 57]. This hypothetical ancestral eukaryote is often called archezoan. The attractive feature of these hypotheses stems from the postulated phagotrophic lifestyle of the archezoa: like extant amoeba, the archezoa would routinely engulf bacteria one of which would eventually turn into the endosymbiont [58, 59]. The problem with the archezoan scenarios is twofold. First, and most obviously, no primary amitochondrial eukaryotes (would-be archezoa) are known. Second, perhaps more controversially, quantitative arguments have been presented that a cell of typical eukaryotic size and complexity is unsustainable without multiple power-producing organelles such as the mitochondria.

The scenarios of the second group are based on the postulate that the cell that captured the endosymbiont was a regular archaeon, and endosymbiosis actually triggered eukaryogenesis, including the emergence of the endomembrane system and other signature attributes of eukaryotic cells [10, 38, 60]. These symbiogenetic scenarios do not assume any unknown ancestral cell types, and arguably credible causative chains have been proposed for the origin of the eukaryotic cellular organization. The weakness of these scenarios is in the apparent extreme rarity of endosymbiosis among bacteria and archaea.

Could there be a third way that would combine the advantages of the two types of scenarios while avoiding the drawbacks of each? I address this possibility in the discussion that follows.

## The scattered archaeal ‘eukaryome’ and the possibility of a complex archaeal ancestor of eukaryotes

Recent analysis of diverse archaeal genomes resulted in a series of striking observations. It turns out that the evolutionary relationship between archaea and eukaryotes is not limited to the core of information-processing systems but also involves several genes and entire gene suites that are essential for eukaryotic intracellular organization [61]. Surprisingly, however, these homologs of the signature eukaryotic genes are scattered among different archaea. Perhaps the most notable case is the ubiquitin system that has been identified in the single sequenced genome from the new phylum Aigarchaeota, *Candidatus Caldiarchaeum subterrenium* [31]. Ubiquitin-like proteins and the ubiquitin-conjugating machinery have been previously identified in other archaea but these were distant homologs of the respective eukaryotic proteins, so the ancestral relationship remained unclear [62, 63]. The case of *C. subterrenium* is different. In this genome, the genes for a ubiquitin homolog, ubiquitin ligase and a key deubiquitinase form an operon, and most important, in the respective phylogenetic trees, these proteins clearly cluster with the eukaryotic homologs. Thus, there is little doubt that the archaeal ancestry of the ubiquitin systems has been traced. Equally consequential is the discovery of archaeal actins (dubbed crenactins) that are present in several groups of Proteoarchaeota [64] and have been shown to form filaments resembling the eukaryotic cytoskeleton [65]. Other examples of apparent archaeal ancestors of key eukaryotic systems involved in the formation of intracellular structures are tubulins [66] and the ECSRT-III complex that participates in cell division and intracellular membrane remodeling [67, 68]. Notably, these signature genes were found mostly in different groups of Proteoarchaeota, in accord with the latest phylogenomic results discussed above.

The discovery of this scattered “archaeal eukaryome” has prompted the hypothesis of a complex archaeal host for the protomitochondrial endosymbiont . Given the extensive horizontal gene transfer in archaea combined with the observations that most archaeal lineages apparently evolved under a streamlining regime [40, 69], it has been speculated that this ancestral archaeal form combined, within a single genome, various components of the eukaryome that are scattered among the extant archaea. This hypothetical organism, although distinctly archaeal, might have been capable of a primitive form of phagocytosis which would facilitate the capture of the endosymbiont [38, 61, 64]. Conceivably, this ancestral archaeon would actively acquire genes via horizontal gene transfer, thus suggesting an alternative explanation for the different affinities of ‘bacterial’ genes in eukaryotes.

An unexpected recent discovery made by methods of single cell genomics indicates that archaea resembling the putative complex ancestors of eukaryotes are not extinct.

## Loki: archaeal ancestor of eukaryotes found alive and well?

Enter Loki. Metagenomic analysis of sea floor sediments near a hydrothermal vent site in the Arctic named Loki’s Castle has revealed a putative deep archaeal lineage within Proteoarchaeota [1, 2]. Being keenly interested in archaea that potentially could shed light on the origin of eukaryotes, Thijs Ettema and colleagues undertook deep sequencing of the metagenomic samples from Loki’s Castle and succeeded in assembling a nearly complete genome as well as several partial genomes from a new archaeal group they named Lokiarchaeota (simply Loki, for short). The results of the Loki genome analysis exceed the boldest expectations. Indeed, Loki combines the two key features predicted for the archaeal ancestor of eukaryotes by the hypothesis discussed in the preceding section. First, in a phylogenetic tree of 36 highly conserved genes encoding components of information-processing systems, eukaryotes convincingly fall within the Loki branch (Fig. 2). This finding settles the issue of the evolutionary relationship between eukaryotes and archaea: there is no longer any reasonable doubt that the information-processing systems of eukaryotes evolved from a specific branch deep within the archaeal tree, and now that branch is known.

Second, and equally important, the genome of Loki reveals the assortment of the signature eukaryotic features that has been predicted for the archaeal ancestor of eukaryotes [38, 61, 64]. Specifically, Loki encodes crenactins, homologs of eukaryotic gelsolins (another family of essential cytoskeleton proteins), the ESCRT-III complex, an expanded family of small Ras-like GTPases and the complete ubiquitin system. This gene repertoire translates into a confident prediction of a complex cytoskeleton and membrane remodeling systems and is compatible with a rudimentary phagocytic capability. Moreover, phylogenetic analysis indicates that most of these homologs of signature eukaryotic genes occupy the basal position in the respective trees, adding credence to the ancestral relationship [1].

Thus, Loki is by far the best current candidate for the role of a direct descendant of the archaeal ancestor of eukaryotes. It is crucial to emphasize that, all its genomic and predicted organizational complexity notwithstanding, Loki is a typical archaeon and not the hypothetical archezoan. Despite the presence of elements of cytoskeleton, key features that are readily detectable in any eukaryotic genome, such as components of the nuclear pore and the spliceosome, as well as spliceosomal introns, are missing, and the entire replication machinery as well as the suite of membrane biogenesis enzymes all have telltale archaeal features [1]. Thus, although the discovery of Loki falls short of eliminating the archezoan scenario of eukaryogenesis once and for all, it substantially increases the credibility of the symbiogenetic scenario.

## Implications and remaining open questions

The extremely hard problem of eukaryogenesis now appears perceptibly more tractable thanks to the advances of comparative genomics of archaea and in particular the spectacular progress of metagenomics. The path to this new understanding was paved by the sequencing of many diverse archaeal genomes followed by detailed phylogenomic analysis. These efforts produced mounting evidence of the evolutionary relationship between Proteoarchaeota and Eukaryota, and enabled the partial reconstruction of the genome of a complex archaeal ancestor of eukaryotes. The discovery of Loki precipitated the breakthrough. The origin of eukaryotes from a specific group of archaea, lodged deep within the archaeal evolutionary tree and specifically within Proteoarchaeota, now should be considered an established fact. Moreover, we also know that the closest extant archaeal relatives of eukaryotes encode a variety of likely ancestors of signature eukaryotic genes that contribute to the cytoskeleton and other aspects of eukaryotic cellular organization. These observations make the symbiogenetic scenario of eukaryogenesis look more credible than it ever did in the past.

The newly achieved clarity in our understanding of these key aspects of eukaryogenesis calls for reassessment of some of the most general concepts in biology. The first one is the representation of the entire history of life as a single evolutionary tree, a grand idea that goes back to the famous single illustration of Darwin’s *Origin of Species* [70]. The symbiogenetic scenario of eukaryogenesis flatly defies this concept because under this scenario, a major kingdom of life, the eukaryotes, emerged in a non-tree-like manner, through fusion of different, distant branches of the tree. The importance of trees for understanding the evolution of individual genes, gene ensembles and major taxa, especially those that encompass multicellular eukaryotes, is undeniable [71]. However, the new findings on the origin of eukaryotes as well as the origin of archaeal phyla [72] indicate that major transitions in evolution often, perhaps typically, occur through the fusion of cells and/or genomes of distantly related organisms. The second, not unrelated general theme is the number and nature of the primary domains of life. In the late 1980s, based on the rRNA trees, Woese and colleagues developed the three-domain scheme (Fig. 1a) [19]. The placement of eukaryotes within the archaeal branch that has been clinched by the discovery of Loki refutes this scheme and shows that the only consistent interpretation of the phylogeny of the universal (primarily informational) genes involves two primary domains: bacteria and archaea (with eukaryotes included) (Fig. 1b) [45].

Loki is named after the trickster god of Norse mythology. He is supposed to have a malicious streak about him but he is also the harbinger of change [73]. Surely, the transition that Loki or his relative seems to have brought about, the origin of eukaryotes, was one of the most momentous in the history of our planet.

What next? Does Loki bridge archaea and eukaryotes as stated in the title of the article by Ettema and colleagues? I think this is still only a halfway bridge. A lot of difficult work remains to be done to join the two banks. First, Loki certainly is not the archaeal ancestor of eukaryotes: that life form existed over a billion years ago. It is entirely possible and actually likely that even closer relatives of eukaryotic ancestors may be discovered, perhaps with an even greater organizational complexity. Loki is only the beginning of the quest for those ancestors, by no means the end. However, further, even possibly exhaustive characterization of archaeal (and bacterial) diversity by methods of metagenomics and single cell genomics is the easy part of the deal. The challenge lies in the investigation of the biology of these organisms. Although we can never know what precisely happened more than a billion years ago, to me, demonstration of the archaeal–bacterial endosymbiosis in the laboratory would mean the completion of the bridge. This is an extremely tall order but then again, who would have predicted 25 years ago that complete genome sequencing of microbes that do not grow in culture would become a near routine exercise?
